# Leflunomide-Induced Drug Reaction With Eosinophilia and Systemic Symptoms: A Diagnostic Dilemma

**DOI:** 10.7759/cureus.72287

**Published:** 2024-10-24

**Authors:** Anjali Jayakumar, Harikrishnan S, Tomy Philip, Meenu Rose Jomey

**Affiliations:** 1 General Medicine, Pushpagiri Institute of Medical Sciences and Research Centre, Tiruvalla, IND; 2 Gastroenterology, Pushpagiri Institute of Medical Sciences and Research Centre, Tiruvalla, IND

**Keywords:** dress, drug reaction, leflunomide, liver biopsy, rheumatoid arthritis

## Abstract

Drug reaction with eosinophilia and systemic symptoms (DRESS) syndrome is a severe idiosyncratic drug reaction. This commonly manifests with fever, lymphadenopathy, and a maculopapular rash. It can be fatal if early diagnosis and removal of the offending agent do not occur. Leflunomide, a disease-modifying antirheumatic drug (DMARD) has been rarely associated with DRESS syndrome. Here, we present the case of a 59-year-old male patient admitted with leflunomide-induced DRESS syndrome.

## Introduction

Drug rash with eosinophilia and systemic symptoms (DRESS) syndrome is a severe idiosyncratic drug reaction with a long latency period. This syndrome usually presents anywhere from two to eight weeks after initiating the offending drug. It is followed by various clinical manifestations, usually fever, rash, lymphadenopathy, eosinophilia, and a wide range of mild-to-severe systemic presentations [1]. The diagnostic criteria proposed by the International Registry of Severe Cutaneous Adverse Reactions (RegiSCAR) help establish the diagnosis [1]. The estimated incidence of DRESS in the general population is more than 1 case per 10,000 exposures to medications. The incidence ranges from 2.18 to 40/100 000 inpatients in hospitalized patients. Despite treatment, the mortality rate in DRESS can range from 3.8% to 10% [2]. While the precise cause of DRESS remains unknown, three major factors have been identified as contributing to its pathophysiology, namely, a genetic predisposition to specific HLA alleles; a change in the drug's metabolic pathways, primarily for aromatic anticonvulsants and, lastly, the reactivation of HHV, which triggers an inflammatory response mediated by T cells and consequent tissue damage [1,2]. Leflunomide, a disease-modifying and antirheumatic drug (DMARD), has been very rarely reported as a cause of DRESS syndrome [3]. Characteristically, DRESS has a latent period of two to eight weeks [4].

## Case presentation

A 59-year-old male patient presented to the emergency department in a tertiary care hospital in Central Kerala with complaints of generalized itching and yellowish discoloration of eyes in the last two days before admission associated with lethargy and one episode of vomiting. These symptoms were preceded by high-grade fever lasting for a day. Further details revealed a weight loss for one year (8 kg) and loss of appetite for two weeks. His past medical history included systemic hypertension, coronary artery disease on antiplatelets and statins, chronic kidney disease on medical management, hyperuricemia on febuxostat, and rheumatoid arthritis on treatment. One month back, he was initiated on leflunomide. He was a non-smoker and did not have a history of alcohol consumption. On examination, he was conscious and oriented, febrile (100.4 °F), pulse rate was 98 beats/min, and blood pressure was 110/60 mmHg. The patient looked icteric. Bilateral discrete, multiple inguinal lymph nodes (1-2 cm) were also palpable. Excoriations were present over arms. The liver and spleen were not palpable. He was admitted with a provisional diagnosis of viral hepatitis/cholestatic jaundice, and baseline investigations were sent. Complete blood count showed hemoglobin of 10.2 gm/dL, total leucocyte count of 6400 with polymorphic predominance (polymorphs - 90, lymphocytes - 6, eosinophils - 2, monocytes - 2), platelet count of 2.5 lakh, and elevated ESR of 60 mm/hr. Renal function tests were deranged (urea - 105 mg/dl, creatinine - 3.3 mg/dl), and liver function test (LFT) showed conjugated hyperbilirubinemia with elevated enzymes (total (T.) bilirubin - 3.7 mg/dl, direct bilirubin - 2.4 mg/dl, serum glutamic-oxaloacetic transaminase (SGOT) - 108 IU/L, serum glutamic pyruvic transaminase (SGPT) - 138 IU/L, alkaline phosphatase (ALP) - 454 IU/L, gamma-glutamyl transferase (GGT) - 277 IU/L). Chest X-ray showed no significant abnormality. ECG showed a first-degree heart block. His coagulation profile and serum electrolytes were within normal limits. The urine routine revealed no traces of any bile salt or bile pigments.

However, ultrasound and subsequent CT abdomen showed no evidence of intrahepatic biliary radicle dilatation. Endoscopic ultrasound (EUS) showed no evidence of obstructive jaundice. Leptospira polymerase chain reaction (PCR), dengue profile, Scrub typhus immunoglobulin M (IgM), hepatitis B surface antigen (HBsAg), hepatitis A IgM, and hepatitis C virus (HCV) chemiluminescent immunoassay (CLIA) test were negative. Statins and febuxostat were stopped given the deranged liver function tests. After sending blood and urine culture sensitivity, the patient was started empirically on antibiotics and other supportive measures. IgM anti-HBc (core antibody), an antinuclear antibody, and an indirect immunofluorescence assay (to rule out autoimmune hepatitis and primary biliary cholangitis (PBC)) were also sent.

**Figure 1:**
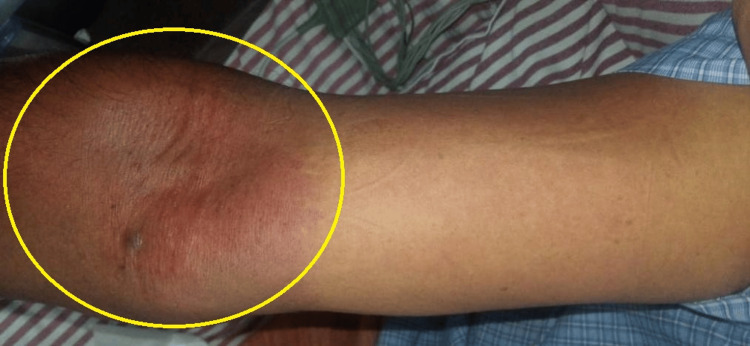
Maculopapular rash over the right forearm

On the fourth day of admission, he developed oral ulcers with a burning sensation and inability to swallow and a maculopapular rash over the upper limbs spreading to the trunk (Figure 1). Temperature monitoring showed persistent fever spikes (up to 103°F), and despite treatment, his itching worsened along with a further rise in bilirubin and liver enzymes (Table 1).

**Table 1:** Worsening trends in liver function tests (LFTs) from the day of admission

LFT	Day 1	Day 2	Day 3	Day 4
Total bilirubin (mg/dl)	3.86	4.27	5.03	5.5
Conjugated bilirubin (mg/dl)	2.6	3	3.3	3.7
SGOT (serum glutamic-oxaloacetic transaminase) (IU/L)	90	110	116	187
SGPT (serum glutamic pyruvic transaminase) (IU/L)	123	140	156	175
Alkaline phosphatase (IU/L)	446	481	549	592

It was also noticed that the eosinophilic count kept increasing in subsequent complete blood count panels (Table 2). The peripheral blood smear showed mild normocytic normochromic anemia and moderate eosinophilia. The absolute eosinophil count (AEC) was 100.

**Table 2:** Increasing eosinophil counts in complete blood counts gm/dL: grams per deciliter; µL: microlitre

CBC	DAY-1	DAY-2	DAY-3
Hemoglobin (gm/dL)	10.2	10.5	10.1
Total leukocyte count (cells/µL)	6400	6500	8800
Neutrophils (cells/µL)	90	72	60
Lymphocytes (cells/µL)	6	4	6
Eosinophils (cells/µL)	2	20	31
Basophils (cells/µL)	2	4	3

**Figure 2:**
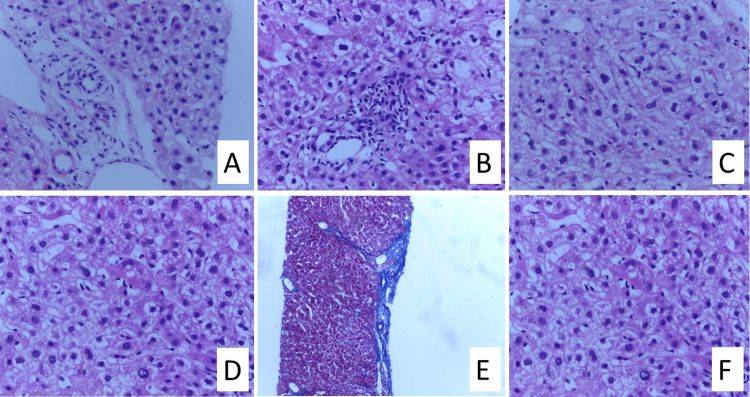
(A) Hepatocyte ballooning with feathery degeneration around the portal triad; (B) Patchy lobular inflammation; (C) Binucleated forms; (D) Glycogenated nuclei; (E) Bridging fibrosis; (F) Bile stasis with eosinophils The regions of importance are focused on in the slides.

Based on the above observations and investigations, we considered four etiological causes: granulomatous liver disease, drug reaction with eosinophilia and systemic symptoms (DRESS), autoimmune hepatitis, and primary biliary cirrhosis (PBC), out of which the most likely possibility was that of DRESS syndrome. Hence, given the possible hepatotoxicity, leflunomide was stopped. Blood culture was suggestive of skin colonizer, and urine cultures were sterile. However, the hepatitis B core IgM antibody was positive at 30 (high) along with antinuclear antibody (ANA) immunofluorescence assay (IFA), which was positive for the homogenous pattern. Upon further evaluation, HBV DNA viral load and HBsAg were negative. This created a diagnostic dilemma. Hence, we decided to proceed with a transabdominal liver biopsy (Figure 2), which showed patchy lobular infiltrates with lymphocytes and bile stasis with eosinophils, suggesting a drug-induced liver injury. The International Registry of Severe Cutaneous Adverse Reactions (RegiSCAR) Group Diagnostic Scoring system scored 6, indicating a definite case of DRESS (Table 3).

**Table 3:** RegiSCAR criteria RegiSCAR: International Registry of Severe Cutaneous Adverse Reactions

Criteria	No	Yes	Unknown	Case 1
Fever >38°C	-1	0	-1	0
Enlarged lymph nodes (>2 sites, >1 cm)	0	1	0	1
Atypical lymphocytes	0	1	0	0
Eosinophilia 700-1499 or 10-19.9%	0	1	0	0
>1500 or >20%	0	2	0	2
Skin rash extent >50%	0	1	0	1
At least two: edema, infiltration, purpura scaling	-1	1	0	0
Biopsy suggesting DRESS	-1	0	0	0
Internal organ involved: One	0	1	0	1
Internal organ involved: Two or more	0	2	0	0
Resolution >15 days	-1	0	-1	0
At least three biological investigations done and negative to exclude alternative diagnoses	0	1	0	1
TOTAL SCORE	6			

Antibiotics were stopped, and the patient was started on injection dexamethasone (IV 8 mg/day). Following this, he improved drastically - his appetite increased, LFT improved (Table 4), and his itching and fever spikes subsided. Once his symptoms reduced, he was discharged on oral steroids (T. prednisolone 30 mg OD) with the advice to modify his anti-rheumatic drugs.

**Table 4:** Improving liver function tests once started on steroids

LFT	Day 20	Day 24	Day 26	Day 29
Total bilirubin (mg/dl)	6.5	5.31	4.1	3.5
Conjugated bilirubin (mg/dl)	4.4	3.3	2.5	2
SGOT (serum glutamic-oxaloacetic transaminase) (IU/L)	55	31	30	29
SGPT (serum glutamic pyruvic transaminase) (IU/L)	86	37	27	25
Alkaline phosphatase (IU/L)	460	411	406	395

## Discussion

DRESS syndrome is a severe, drug-induced, idiosyncratic multisystem reaction to a drug, characterized by fever, skin rash, lymphadenopathy, hematological abnormalities, and internal organ involvement. Characteristically, DRESS has a latent period of two to eight weeks [4]. Leflunomide (LEF) is not usually associated with DRESS syndrome. To treat active rheumatoid arthritis, physicians provide LEF, a disease-modifying antirheumatic medication (DMARD). The most often reported side effects of LEF therapy are gastrointestinal problems, such as nausea and diarrhea, whereas DRESS syndrome is less common. To the best of our knowledge, only nine cases of LEF-induced DRESS syndrome have been documented in the past, according to a PubMed literature review, indicating that the rate of LEF-induced DRESS syndrome is much lower than the typical frequency [5]. Those most frequently implicated are aromatic anticonvulsants (phenytoin, carbamazepine, and phenobarbital), sulfonamides, sulfones (dapsone), nonsteroidal anti-inflammatory drugs, beta-lactam antibiotics, vancomycin, allopurinol, minocycline and antiretrovirals [6].

Numerous clinical and analytical markers, as well as RegiSCAR criteria, are used in diagnostic grading. A delayed hypersensitivity reaction, in which the offending drug particularly activates CD4 and CD8 T cells to overproduce cytokines and acute phase reactants, is one of the various causes causing DRESS that have been proposed. Another idea holds that the underlying Epstein-Barr virus, or CMV, is activated by the offending agent, and the body's immune system attacks the virus, causing subsequent symptoms [7]. First and foremost, fluid resuscitation, symptom control, and stopping the offending substance are critical. Oral prednisone is advised for a moderate illness that does not manifest as overt organ damage. Patients with severe DRESS syndrome who have one or more end-organ involvement should consider oral steroids, systemic steroids, or IV methylprednisolone dosed at 1 mg/kg with a two- to three-month taper. Intravenous immunoglobulins and cyclosporine are two other treatment modalities. Leflunomide-associated DRESS indicates cholestyramine wash-out therapy in which cholestyramine-facilitated removal of the drug occurs [8]. The average time to recovery is six to nine weeks. In the long term, most patients do well, although some patients can go on to develop autoimmune diseases, so additional monitoring should be considered [9,10]. In this case, the patient also showed good recovery upon a three and six-week follow-up; hence, the medications were tapered accordingly.

## Conclusions

A rare condition known as DRESS syndrome can be lethal and arises from an adverse pharmaceutical reaction. This case illustrates the need for doctors to be aware of the challenges in correctly diagnosing DRESS syndrome due to its overlap with several different hepatobiliary and rheumatological illnesses, particularly when it manifests as an extremely unusual adverse drug reaction.
